# Decision tree-based learning to predict patient controlled analgesia consumption and readjustment

**DOI:** 10.1186/1472-6947-12-131

**Published:** 2012-11-14

**Authors:** Yuh-Jyh Hu, Tien-Hsiung Ku, Rong-Hong Jan, Kuochen Wang, Yu-Chee Tseng, Shu-Fen Yang

**Affiliations:** 1 Institute of Biomedical Engineering, National Chiao Tung University, Hsinchu, Taiwan; 2 Department of Computer science, National Chiao Tung University, Hsinchu, Taiwan; 3 Department of Anesthesiology, Changhwa Christian Hospital, Changhwa, Taiwan

**Keywords:** Classification, Data cleaning, Decision tree-based learning, Pain management, Patient Controlled Analgesia (PCA)

## Abstract

**Background:**

Appropriate postoperative pain management contributes to earlier mobilization, shorter hospitalization, and reduced cost. The under treatment of pain may impede short-term recovery and have a detrimental long-term effect on health. This study focuses on Patient Controlled Analgesia (PCA), which is a delivery system for pain medication. This study proposes and demonstrates how to use machine learning and data mining techniques to predict analgesic requirements and PCA readjustment.

**Methods:**

The sample in this study included 1099 patients. Every patient was described by 280 attributes, including the class attribute. In addition to commonly studied demographic and physiological factors, this study emphasizes attributes related to PCA. We used decision tree-based learning algorithms to predict analgesic consumption and PCA control readjustment based on the first few hours of PCA medications. We also developed a nearest neighbor-based data cleaning method to alleviate the class-imbalance problem in PCA setting readjustment prediction.

**Results:**

The prediction accuracies of total analgesic consumption (continuous dose and PCA dose) and PCA analgesic requirement (PCA dose only) by an ensemble of decision trees were 80.9% and 73.1%, respectively. Decision tree-based learning outperformed Artificial Neural Network, Support Vector Machine, Random Forest, Rotation Forest, and Naïve Bayesian classifiers in analgesic consumption prediction. The proposed data cleaning method improved the performance of every learning method in this study of PCA setting readjustment prediction. Comparative analysis identified the informative attributes from the data mining models and compared them with the correlates of analgesic requirement reported in previous works.

**Conclusion:**

This study presents a real-world application of data mining to anesthesiology. Unlike previous research, this study considers a wider variety of predictive factors, including PCA demands over time. We analyzed PCA patient data and conducted several experiments to evaluate the potential of applying machine-learning algorithms to assist anesthesiologists in PCA administration. Results demonstrate the feasibility of the proposed ensemble approach to postoperative pain management.

## Background

Pain is one of the most commonly reported postoperative symptoms [1]. Pain can negatively affect quality of life and may do more harm than an illness itself when it becomes intolerable, making the patient both physically and mentally uncomfortable. Pain is a highly personal experience influenced by multiple factors, including sensitivity to pain, age, genetics, physical status, and psychological factors [2,3]. Progress in medical science has gradually made people more aware of the importance of pain management.

Patient-controlled analgesia (PCA) is a pain medication delivery system that enables effective and flexible pain treatment by allowing patients to adjust the dosage of anesthetics. According to previous research [4,5], PCA has become one of the most effective techniques for treating postoperative analgesia. As a result, PCA is now widely used in hospitals for the management of postoperative pain, especially for major surgeries.

This study focuses on PCA. Previous research has identified the preoperative correlates of postoperative pain intensity or analgesic consumption in various patient groups of different genders, ages, or psychological states [6,7]. These studies used statistical methods, such as ANOVA, chi-square tests, or regression analysis, to evaluate this correlation in an attempt to identify tailored treatments that reduce severe postoperative pain or improve acute and chronic outcomes. With the same objective, this study applies learning algorithms to predict (1) the postoperative analgesic requirement and (2) the need for PCA setting readjustment (e.g., lockout) based on patient physical states and the first few hours of PCA treatment data.

## Methods

### Subjects in study and goals of prediction

This study was conducted with the approval of the Institutional Review Board at Changhwa Christian Hospital (CCH). PCA usage profiles from 2005 to 2010 were collected for analysis. The Abbott Pain Management Provider (Abbott Lab, Chicago, IL, USA) was used for PCA treatment. Instructions were reviewed with patients before receiving PCA therapy. With the assistance of the Acute Pain Service, more than 5000 patient records dated from 2005 were retrospectively collected. After discarding incomplete PCA log files and patient records with missing demographic, biomedical, or surgery-related attributes, we obtained 2,207 patient records. Of these patients, 1,108 were excluded from the sample because their PCA medication was administered for less than 72 h. This is because this study focuses on patients that received at least 72 h of PCA treatment. Thus, the final sample included 1,099 participants after data preprocessing. Table 1 presents a summary of their attributes, which were divided into four categories: (a) patient demographic attributes, (b) biomedical attributes, (c) operation-related attributes, and (d) PCA-related attributes. Attribute values were either nominal or numeric. Given the physical states of patients and their first 24-h PCA treatment profiles, we predicted (a) the total anesthetic dose taken in subsequent hours, and (b) whether any PCA control, (e.g., lockout time or PCA dosage) should be readjusted. This study has two main goals. First, based on accurate prediction, we hope to provide an early warning for anesthesiologists to make necessary changes in analgesic dosage or PCA control settings to improve patient satisfaction during postoperative pain management. Second, based on comprehensible prediction, this study attempts to identify significant factors that affect analgesic requirement.

**Table 1:** Summary of patient attributes

**Attribute Name**	**Description**
**Demographic:**	
***age***	patient age
***gender***	patient gender
***weight***	patient weight
**Biomedical:**	
***pulse***	heart rate
***sbp***	systolic blood pressue
***dbp***	diastolic blood pressure
***DM***	if patient is diabetic
***HT***	if patient has hypertension
***AMI***	if patient has acute myocardial infarction
ASA_CLASS^*^	1: healthy
	2: mild systemic disease
	3: major systemic disease
	4: life-threatening disease or condition
	5: not expected to survive
	6: donor
**OP-related:**	
**OP_CLASS**	surgical type:
	1: intrathoracic
	2: upper intra-abdominal
	3: lower intra-abdominal
	4: laminectomy
	5: major joints
	6: limbs
	7: head& neck
	8: others
**op_time**	surgical duration
**URGENCY**	E: emergency surgery
	R: regular surgery
**ANS_WAY**	SA: spinal anesthesia
	GA: general anesthesia
	LE: lumbar epidural anesthesia
	NB: nerve blockade
**PCA-related:**	
**loading_dose**	analgesia taken before PCA treatment
**sucess_p_1hr~ sucess_p_24hr**	number of successful PCA demands in 1^st^–24^th^ h
**failure_p_1hr~ failure_p_24hr**	number of PCA demands that fail in 1^st^–24^th^ h
**pcadose_1hr~ pcadose_24hr**	total PCA dose in 1^st^–24^th^ h
**contidose_1hr~ contidose_24hr**	total continuous dose in 1^st^–24^th^ h
**readjustcount_1hr~ readjustcount_24hr**	number of PCA readjustment in 1^st^–24^th^ h
**p_timediff_mean_1hr~ p_timediff_mean_24hr**	mean of time gap between two consecutive PCA demands
**p_timediff_var_1hr~ p_timediff_var_24hr**	variance of time gap between two consecutive PCA demans
**pcamode_set_1hr~ pcamode_set_24hr**	setting of PCA mode:
	(a) PCA and continuous
	(b) PCA only
**pcadose_set_1hr~ pcadose_set_24hr**	PCA dose setting in 1^st^–24^th^ h
**lockout_set_1hr~ lockout_set_24hr**	setting of minimum time gap between two adjacent PCA demands in 1^st^–24^th^ h
**4hrlimit_set_1hr~4hrlimit_set_24hr**	setting of maximum dosage allowed for every 4 h in 1^st^–24^th^ h

^*^ASA class is the commonly used preoperative index of physical status defined by the American Society of Anesthesiologists.

### Analgesic consumption prediction

Some researchers have used regression analysis to derive predictive models of analgesic requirements or postoperative pain [7-9]. Although they identified several positive correlates, such as age and gender, their coefficients of determination were small. For example, the best predictor in an analysis of total analgesic need was the State Trait Anxiety Inventory, but its coefficient was only 0.22 [9]. This result indicates the limitations of regression analyses and suggests that other predictive factors are present that have not been analyzed. This study includes PCA-related factors in addition to demographic and physiological attributes. Unlike approaches that fit the numeric values of analgesic requirements, this study categorizes analgesic consumption into a number of symbolic values (e.g., “small,” “medium,” and “large”). Instead of a numeric value, we tried to predict a symbolic value of analgesic consumption because this indicator is expressive enough for medical staff or anesthesiologists to recognize an abnormality in PCA medications. The discretization of numeric values can also reduce the computational complexity of prediction. To discretize analgesic consumption, the numeric value was divided into several intervals, with each interval corresponding to a specific symbolic value. This process was accomplished by an iterative optimization procedure that identified the intervals and ensured that dose deviations in all intervals were approximately equal.

Prediction methods can be compared and evaluated based on accuracy and comprehensibility. The accuracy of a predictor refers to its ability to correctly predict the value of the target attribute (e.g., total anesthetic dose) for previously unseen data. The comprehensibility of a predictor refers to the level of ease with which people can interpret the predictions. For any prediction method, inductive bias causes some trade-offs between these two criteria [10]. A predictor that can make both accurate and comprehensible predictions is most desirable, but unfortunately, finding a predictor that achieves both accuracy and comprehensibility is difficult and unlikely. Therefore, before development, it is necessary to examine the application domain of the predictor to determine what information users expect the predictor to deliver. For example, an investor making a quick, one-time investment may wish to know only if the stock market will go up or down during the next few weeks. In this case, an answer as simple as “up” or “down” is sufficient, and the user’s primary concern is prediction accuracy. Conversely, for a long-term investment, an investor may require not only an accurate prediction, but also an explanation. With a comprehensible predictor that is easily communicated to the user, predictions can be easier to interpret and verify. The goal of this study is to develop a prediction tool that can make predictions about PCA analgesic requirements with high accuracy and acceptable comprehensibility for anesthesiologists.

Decision tree learning is among the most widely used and practical methods of inductive inference [11]. This method approximates the function for the target attribute by learning a decision tree from previous examples. Each internal node in a decision tree specifies an attribute test, and each leaf represents the predicted target value. If we represent each example by a set of descriptive attributes and its target attribute and their attribute values, then we can define decision tree inductive learning as follows.

Given:

***E*={*e***_***1,***_***e***_***2***_***,…,e***_***n***_**}**: a set of training examples

***X*={*x***_***1,***_***x***_***2***_***,…,x***_***m***_**}**: a set of descriptive attributes

***c***: the target attribute

Each training example *e*_*i*_ is represented by a vector<*v*_*1*_*,v*_*2*_*,…,v*_*m,*_*t*_*i*_>, where *v*_*1*_*,v*_*2*_*,…,v*_*m*_ denotes a legal value of attribute *x*_*1,*_*x*_*2*_*,…,x*_*m*_, and *t*_*i*_ is a legal value of the target attribute *c*.

Assuming:

***F*::*X*→*c***: the target attribute function, which maps an example represented by a vector of descriptive attribute values to its target attribute value.

Learn:

***T*::*X*→*c***: a decision tree that approximates the target attribute function *T*(*X*)≈*F*(*X*).

**Figure 1:**
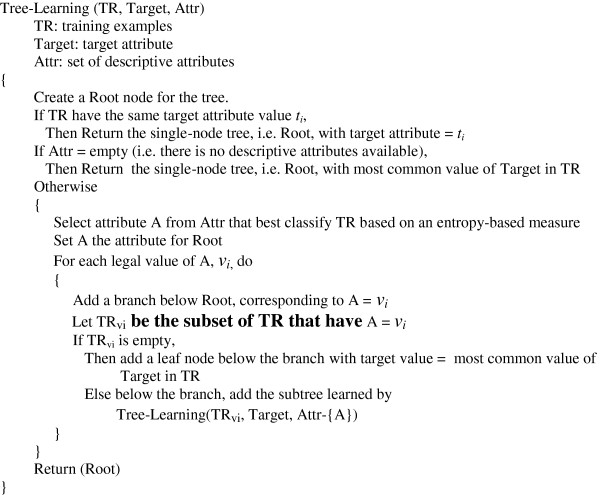
Pseudocode of decision tree learning.

Most decision tree-based learning algorithms are based on a principle algorithm that performs a top-down, recursive greedy search for the best decision tree. This process selects one attribute at a time from the available descriptive attributes as a node in the tree. A descendant of the node is created for each legal value of this attribute. This process is repeated for the training examples associated with each descendant to select the next node in the tree. Figure 1 presents a pseudocode of the principle algorithm, which builds a decision tree in a recursive fashion, and returns its root at last.

**Figure 2:**
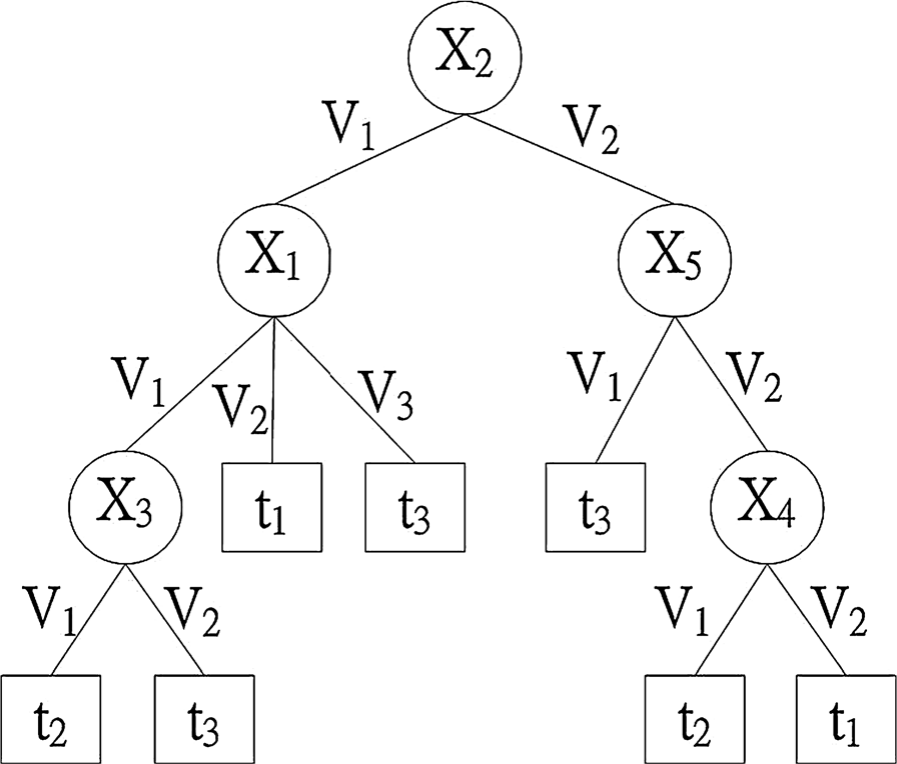
A sample decision tree.

Figure 2 shows an example of decision trees learned by the decision tree learning algorithm. When predicting the target value for a previously unseen example, traverse the learned decision tree from the root according to the descriptive attribute values of the new example until reaching a leaf, which predicts the target attribute value. For example, a new example<*v*_*1*_*,v*_*2*_*,v*_*2*_*,v*_*1*_*,v*_*2*_*,v*_*3*_> has a predicted target value of *t*_*2*_.

Compared with other inductive learning methods (e.g., Artificial Neural Network [12], Support Vector Machine [13], Naïve Bayesian classifier [14]), decision tree learning is more interpretable by humans because a decision tree is a pictorial representation that can be easily translated into a set of if-then-else rules. For example, the left-most path from the root to the leaf in Figure 1 can be translated into “If *x*_*2*_ is *v*_*1*_, *x*_*1*_ is *v*_*1*_, and *x*_*3*_ is *v*_*1*_, then target is *t*_*2*_.” In addition, because the attributes appearing at higher levels in a decision tree are considered more informative [11], a tree can identify significant attributes for further analysis more easily than a model learned by other approaches (e.g., the conditional probabilities of a Naïve Bayesian classifier or the tuned weights of an ANN). To maintain sufficient comprehensibility in prediction and analysis, the proposed PCA prediction tool is based on decision tree learning. This study demonstrates how to explore the resulting decision trees by analyzing the patient attributes used in the resulting trees.

Although decision tree learning has proved useful in many real-world applications, such as SKICAT [15], further studies have shown that an ensemble of decision trees is often more accurate than any single tree [16,17]. Bagging [18] and boosting [19] are two popular methods of creating accurate ensembles. Both methods rely on “re-sampling” techniques to obtain different training sets for each predictor in the ensemble. However, previous research indicates that boosting is more prone to overfitting the training data [20,21]. Consequently, the presence of noise causes a greater decrease in the performance of boosting. Therefore, this study uses bagging to create an ensemble of decision trees to better address the noise in medical data.

**Figure 3:**
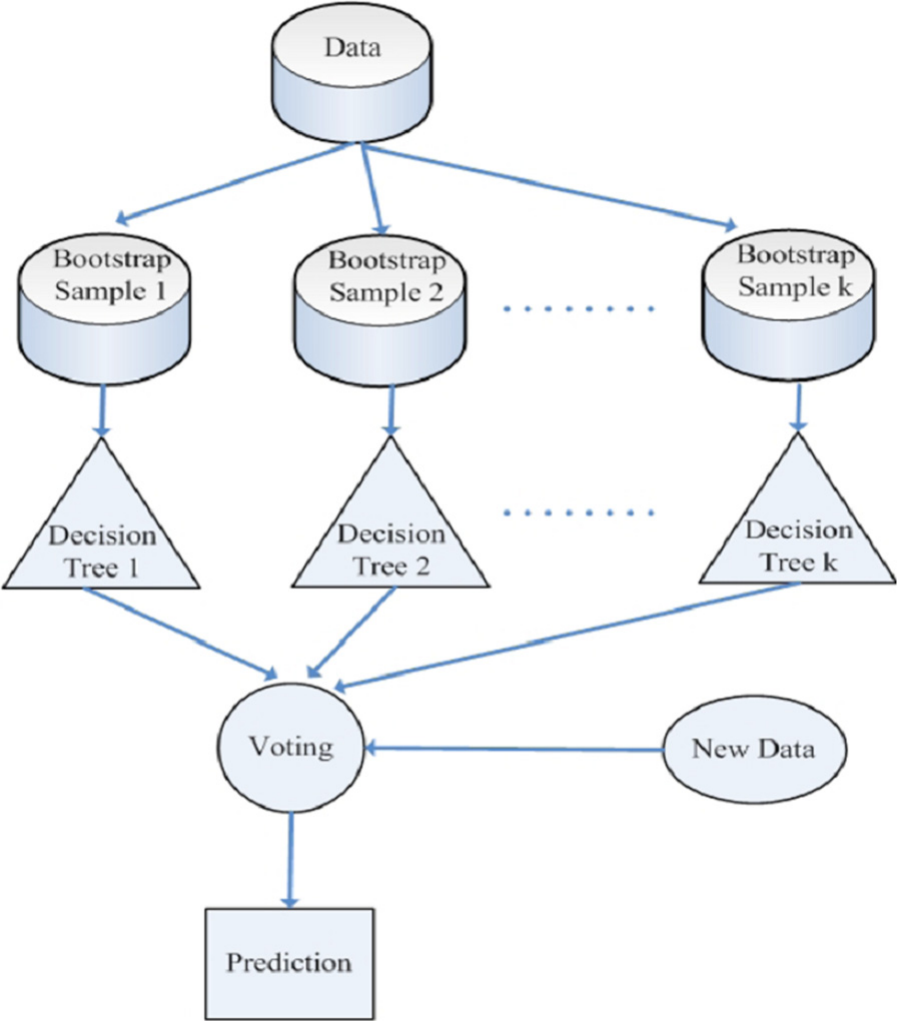
General framework of a bagged decision tree predictor.

Bagging is a method of generating multiple versions of a predictor and combining them to form an aggregated predictor. The idea of bagging can be illustrated by an intuitive example. Suppose that a patient wants a diagnosis made based on the symptoms. He (or she) would rather consult with several physicians than only one. The most frequent diagnosis is likely to be the correct diagnosis because a majority vote by a large group of doctors is likely more reliable. To extend the example, substituting one version of a predictor for each doctor produces the bagging predictor. To produce multiple versions of a predictor in an ensemble, bagging creates a training data set to train each predictor. Each training data set is a bootstrap sample created by sampling the given examples uniformly with replacement. Figure 3 shows a general framework of bagging for the decision tree predictor.

### PCA control readjustment prediction

Little research has been done on the prediction of PCA control readjustment. In addition to predicting analgesic consumption, this study attempts to predict whether any PCA control, including PCA dosage, PCA mode, lockout, and 4-h limit, should be readjusted to improve patient satisfaction. This issue is an anomaly-detection problem [22] because only a few patients require PCA readjustment after the initial setting. The large difference in the number of patients who need PCA readjustment and those who do not creates a class imbalance problem. Learning from imbalanced data sets, in which the number of examples of one (minority) class is much smaller than the other (majority), presents a significant challenge to the machine-learning community [23]. Conventional machine-learning algorithms are typically biased toward the majority class, and produce poor predictive accuracy for the minority class. Researchers have proposed various approaches for coping with imbalanced data sets. Guo and Viktor combined boosting and synthetic data to improve the prediction of the minority class [24]. Cardie and Howe weighted examples in an effort to bias the learning toward the minority class [25]. Joshi et al. evaluated boosting algorithms to classify rare classes [26]. Finally, Khalilia et al. combined repeated random sub-sampling with the Random Forest method to overcome the class imbalance problem [27].

**Figure 4:**
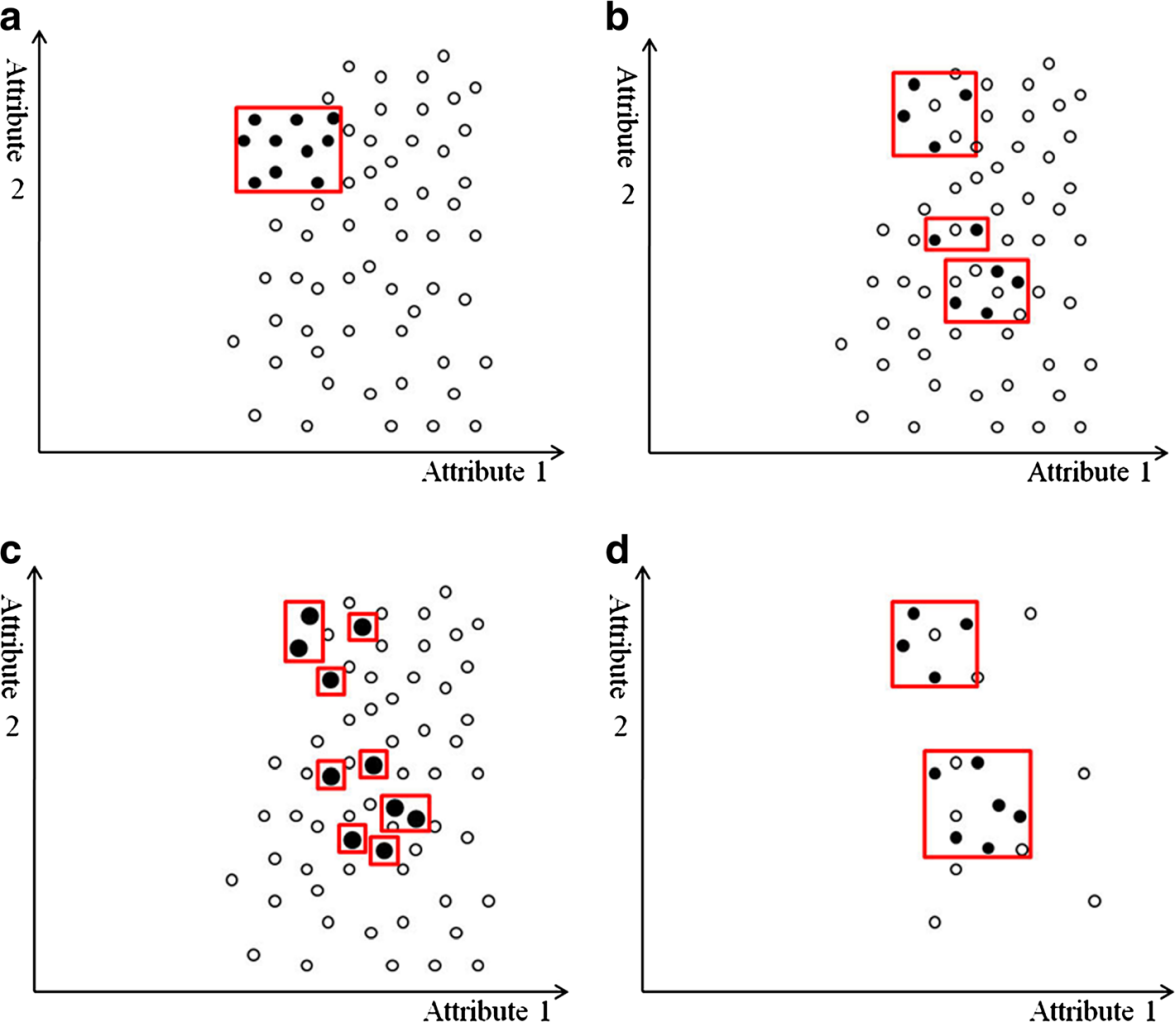
**Examples of decision regions of data points projected to a 2D space.** The X- and Y-axes represent two attributes in the feature space. The minority class examples are denoted by black circles, and the majority class examples are denoted by white circles. Red rectangles indicate the axis-parallel decision regions of the minority class learned by the decision tree algorithm. (**a**) In an imbalanced but coherent data set, the boundary between classes is clear. Over-sampling the minority class or under-sampling the majority class to balance the data set can help learning algorithms identify the decision regions. (**b**) If the data set is imbalanced and the minority class examples are sparsely scattered in the majority class, the decision regions are likely to include the majority class examples, making classification more difficult. (**c**) Over-sampling the minority class with replications makes the decision regions more specific. The replications of the minority class examples are indicated by larger black circles. As the decision regions become more specific, learning algorithms based on the divide-and-conquer method (e.g., a decision tree algorithm) are more prone to overfitting because they produce more partitions in the data during learning. (**d**) In contrast, under-sampling the majority class randomly selects examples until its size equals that of the minority class. Because the minority class examples are scattered, the decision regions may still contain the majority class examples, and learning the boundary remains difficult.

In addition to unequal class distribution, instances sparsely scattered in the data space make the prediction of a minority class even more difficult. If both classes are coherent, as in Figure 4(a), the boundary is clear even if the class distribution is uneven. However, data point sparsity blurs the boundary between classes, as Figure 4(b) shows. Random sampling techniques, such as over-sampling the minority class or under-sampling the majority class, have little effect on improving the boundary. Replicates of the minority class make the decision region of the minority class more specific (Figure 4(c)), and thus cause further splits in decision tree learning [28]. More splits lead to more leaf nodes in a decision tree, and consequently, to a greater tendency to overfitting. Conversely, under-sampling randomly picks examples from the majority class until the number of examples matches the size of the minority class. The examples of the majority class are selected randomly and the examples of the minority class are sparely distributed. Thus, an equal size of both classes does not help show a clearer boundary (Figure 4(d)). The methods that adopt over-sampling by creating artificial minority data or integrating boosting with synthetic data claim to achieve better classification accuracy on the minority class. However, experimental results show that their performance highly depends on the synthetic data created [24,28].

**Figure 5:**
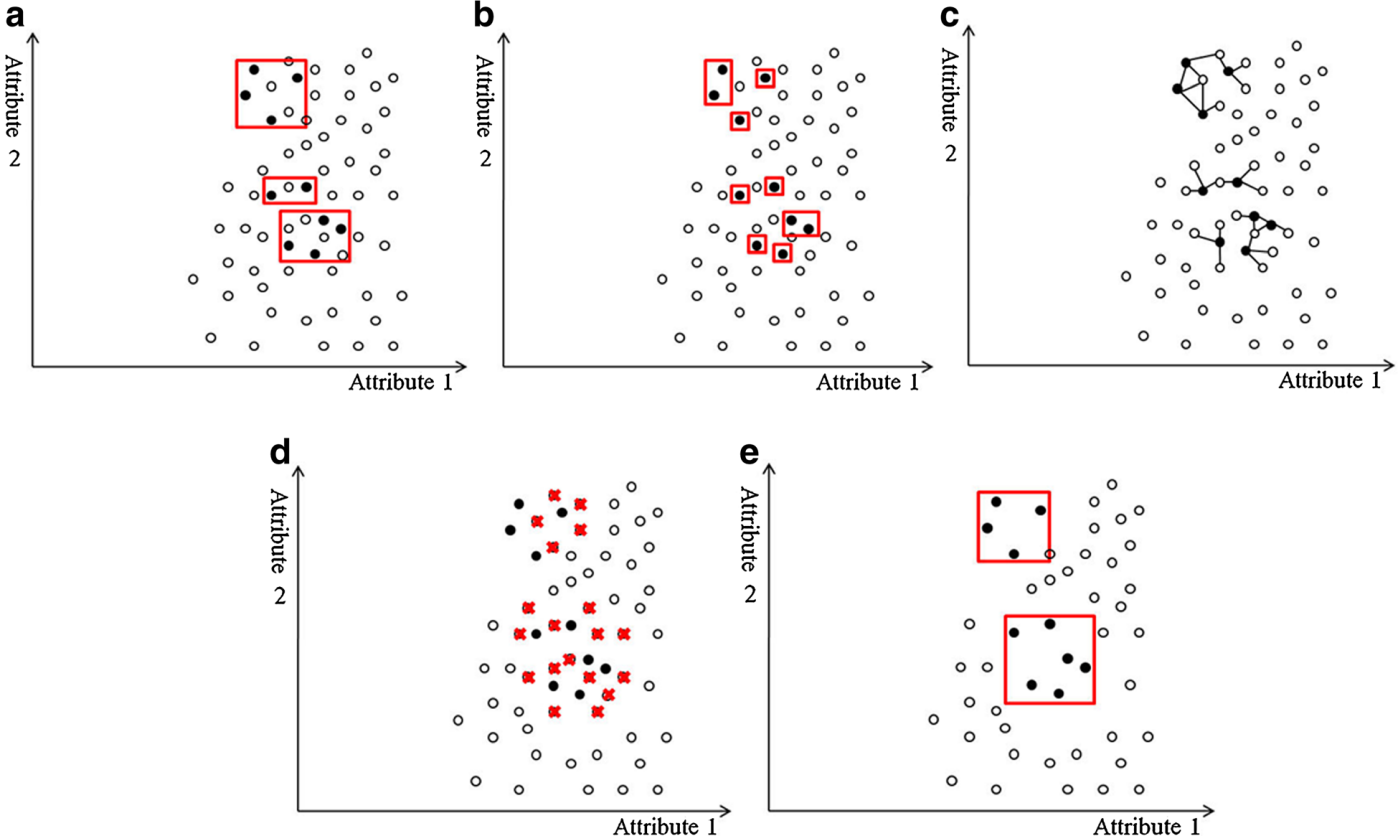
**An example of nearest neighbor–based data cleaning.** The X- and Y-axes represent two attributes in the feature space. The minority class examples are denoted by black circles and the majority class examples are denoted by white circles. Red rectangles indicate the axis-parallel decision regions of the minority class learned by the decision tree algorithm. (**a**) We show an imbalanced data set with sparse minority class examples. The decision regions of the minority class contain the majority class examples. (**b**) One way to exclude the majority class is to shrink the decision regions by making them more specific. However, more specific regions produce more splits in the decision tree, causing the overfitting problem. (**c**) To identify the “dirty” examples that may mislead learning, the proposed method locates *k*-nearest (where *k* is 3 in this example) neighbors for each minority class example. The 3-nearest neighbors of a minority class example are indicated by links. (**d**) A red cross marks each “dirty” example. (**e**) After the “dirty” examples are removed, the decision regions are “clean” (i.e., they contain only the minority class examples). Using these clean decision regions, learning algorithms can more easily recognize the correct boundary between classes.

**Figure 6:**
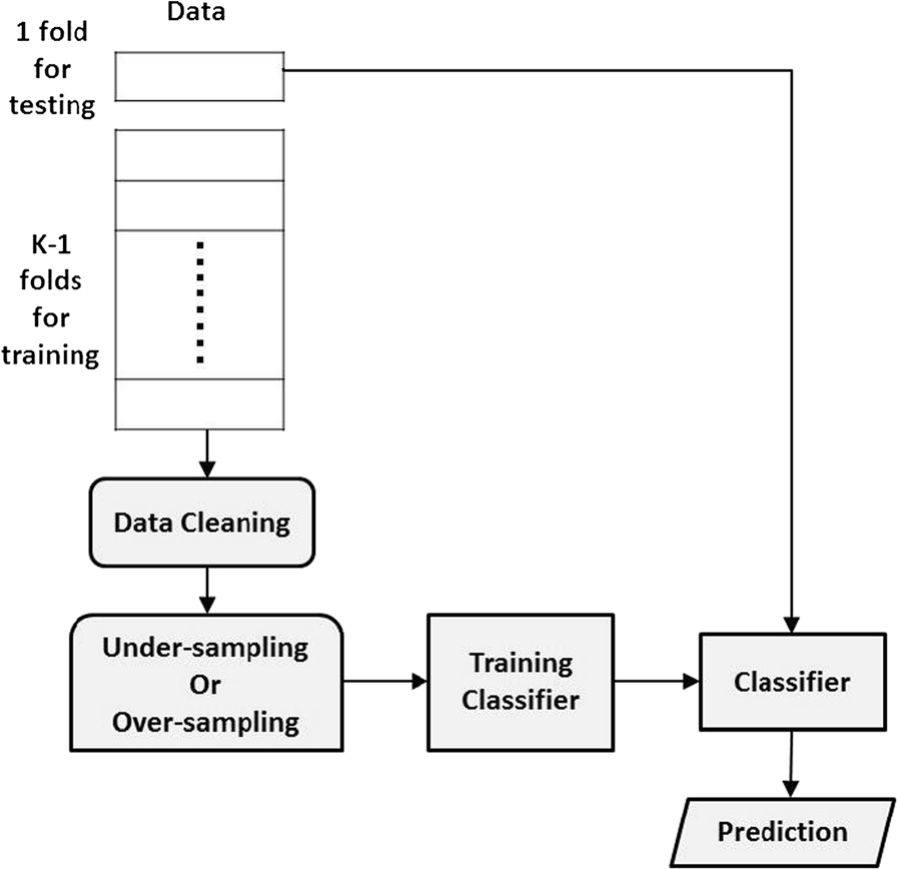
**Control flow of data cleaning, sampling, training and prediction.** This control flow shows only one run in a k-fold cross validation. One fold of the data is used for testing, and the remaining k-1 folds are used for training. To make prediction consistent with the real class distribution, maintain the original class distribution in the test data and only perform data cleaning on the training data. Repeat the same process on each fold of the data as the test data, and use the rest as the training data.

Unlike previous research, the proposed approach combines data cleaning and repeated random sampling techniques to balance data sets. Motivated by the nearest-neighbor approach for outlier detection [29,30], this approach identifies the candidate examples for removal in a neighborhood. Instead of using the distance of a data instance to its *k*th nearest neighbor as an anomaly score [31], this approach first identifies the *k*-nearest neighbors of each instance of the minority class, and considers any majority class neighbor as “dirty.” After examining each instance in the minority class and its neighbors, the proposed approach removes those “dirty” instances. The rationale behind this process is that the nearest majority class neighbors of a minority class member are likely to mislead learning algorithms. Without them, learning algorithms can more easily recognize the minority class boundary. Figure 5 illustrates this concept. Figure 5(a) shows an imbalanced data set before removing “dirty” instances. The rectangles in this figure represent the decision regions of the minority class, and several majority class examples are also included. One way to exclude the majority class examples is to shrink the decision regions, but this shrinkage can lead to overfitting the minority class, as Figure 5(b) shows. Instead, the proposed approach first locates the *k*-nearest neighbors (e.g., *k*=3) for each minority class example, and then presents the neighbors as linked to each minority class example (Figure 5(c)) and crosses out the “dirty” majority class neighbors (Figure 5(d)). Removing the “dirty” examples produces the “clean” decision regions of the minority class (Figure 5(e)). After data cleaning, under-sampling or over-sampling and bagging or boosting techniques can further balance the class distribution. In practice, the number of nearest neighbors (i.e., the value of *k* for *k*-nearest neighbors) is determined by a validation test in which the training data are further divided into two subsets at random. One subset is used to train a classifier after the dirty examples, based on *k*-nearest neighbors, are removed from the subset. The other subset is used to validate the performance of the trained classifier. Varying *k* enables the selection of the *k* that maximizes classifier performance. The proposed data cleaning method is not designed to replace previous approaches to mitigating the class-imbalance problem, but rather to serve as a preprocessor for these approaches. Figure 6 shows the control flow for data cleaning, random sampling, classifier training, and prediction. This study demonstrates the usefulness of data cleaning compared to other approaches tackling class imbalance, including under-sampling, over-sampling, and data generation.

## Results and discussion

### Analgesic consumption prediction results

We predicted the 72-h total analgesic consumption (continuous dose plus PCA dose) for each patient based on the patient’s first 24 h of PCA usage data, physical state, and surgery-related attributes. The numeric value of the total anesthetic dose (continuous and PCA) was discretized into three symbolic values: “low,” “medium,” and “high.” The 1099 patients who received PCA treatment for more than 72 h were divided into three classes according to the symbolic values, with class sizes of 399 (low), 551 (medium), and 149 (high), respectively.

This study evaluates the performance of learning algorithms in analgesic consumption prediction based on predictive accuracy instead of the performance measure used in ordinal classification [32]. This is because some of the learning algorithms applied in the proposed method (e.g., C4.5 decision tree learner) treat “low,” “medium,” and “high” as symbolic values. The performance of learning algorithms was compared based on the conventional predictive accuracy to ensure consistency because this order relationship cannot be used in some of the learning algorithms. The prediction performance of each class was measured separately in addition to the overall accuracy of all classes. Accuracy was calculated according to a confusion matrix, as Table 2 shows for a 3-class prediction problem. In this matrix, the rows represent the predicted classes, and the columns represent the real classes. Each element in the matrix is the number of predictions corresponding to the predicted class and the real class. For example, *a* is the number of *Class*_*low*_ examples correctly classified, and *b* is the number of *Class*_*medium*_ examples misclassified as *Class*_*low*_. In addition to overall accuracy, this study calculates the sensitivity and precision of each class (e.g., *Class*_*low*_ sensitivity was defined as a/(a+d+g)). Table 3 provides a complete description of the performance measures used in these experiments.

**Table 2:** Confusion matrix for analgesic consumption prediction

	**Real Low**	**Real Medium**	**Real High**
***Predicted Low***	a	b	c
***Predicted Medium***	d	e	f
***Predicted High***	g	h	i

**Table 3:** Definitions of performance measures for analgesic consumption prediction

**Performance Measure**	**Definition**
Low Consumption Sensitivity	a/(a+d+g)
Medium Consumption Sensitivity	e/(b+e+h)
High Consumption Sensitivity	i/(c+f+i)
Low Consumption Precision	a/(a+b+c)
Medium Consumption Precision	e/(d+e+f)
High Consumption Precision	i/(g+h+i)
Overall Accuracy	(a+e+i)/(a+b+c+d+e+f+g+h+i)

This study includes a stratified *k*-fold cross-validation experiment to evaluate classifier performance. The initial PCA data (i.e., the 1099 patient records) were randomly divided into *k* disjoint folds (i.e., subsets) of approximately equal size. The folds were also stratified to maintain the same class distribution as in the initial data. One fold of data was used to test the prediction performance, and the remaining (*k*-1) folds were all used for training. The same training–testing process was applied to each fold iteratively. Each run produced a prediction performance result based on the fold selected for testing, and the overall performance consists of the average over all iterations.

Because the goal of this study is to develop an accurate and comprehensible classifier for anesthesiologists, it only applies C4.5 to ensemble learning. This study compares the performance of C4.5 [11] with bagging and boosting in a stratified 10-fold cross validation, and measures the performance in terms of sensitivity and precision of each class and overall accuracy in all classes. Each run of the cross-validation experiment generated 200 bootstrap samples from the training data, creating an ensemble of 200 decision trees. The prediction for a test example was made by taking a majority vote from the bagging trees. The AdaBoost ensemble algorithm [20] was adopted to implement boosting. This study also compares Artificial Neural Network (ANN) [12], Support Vector Machine (SVM) [13], Random Forest [33], Rotation Forest [34], and Naïve Bayesian (NB) classifiers [14].

All learning algorithms were performed for ten iterations of 10-fold cross validation on the same training data set and test data set in each run, and the results were averaged. Table 4 presents the results of a paired *t*-test with Bonferroni correction between bagging and the other methods. These *t*-test results show that the overall accuracy of the bagged C4.5 was significantly better than most of the other methods (*p*< 0.001). In addition to total analgesic consumption prediction (i.e., continuous dose plus PCA dose), this study predicts the total 72-h analgesic consumption exclusively contributed by patient demands (i.e., PCA dose only). Table 5 presents a summary of the results. As in total analgesic dose prediction, the numeric value of PCA analgesic dose was first discretized into three symbolic values: “low,” “medium,” and “high.” The 1099 patients who received PCA treatment for more than 72 h were divided into three classes according to the symbolic values, and the class size was 580 (low), 373 (medium), and 146 (high), respectively. The bagged C4.5 significantly outperformed most of the other methods in predicting PCA analgesic consumption (*p*< 0.001).

**Table 4:** Results of total analgesic consumption (Continuous + PCA) prediction

**Total Analgesic Consum. Prediction (%)**	**C4.5 bagging**	**C4.5 AdaBoost**	**C4.5**	ANN^*^	**Random Forest**	**Rotation Forest**	**SVM**^**‡**^	**NB**
Low Consum. Sensitivity	84.3	79.2	77.4	69.8	80.1	83.1	8.0	79.0
Med Consum. Sensitivity	83.5	75.8	72.8	79.6	83.6	82.0	96.1	67.7
High Consum. Sensitivity	62.4	61.2	60.6	21.6	47.4	62.0	0.0	38.0
Low Consum. Precision	84.3	78.8	76.3	80.2	81.4	82.9	59.4	71.8
Med Consum. Precision	79.7	75.3	73.5	66.1	74.8	78.8	50.6	70.2
High Consum. Precision	78.5	66.0	62.8	56.9	80.4	76.3	0.0	46.3
**Overall Accuracy**	**80.9**	**75.1**	**72.8**	**68.5**	**77.4**	**79.7**	**50.7**	**67.9**

^*^ANN consisting of an input layer of 279 input units, one hidden layer of 140 hidden units, and one output layer of 3 output units.

Learning rate= 0.3; momentum rate= 0.2.

^‡^SVM using a radial basis function, exp(−gamma*|u-v|^2^), where gamma=1/(number of attributes)=1/279.

**Table 5:** Results of PCA analgesic consumption (PCA only) prediction

**PCA Analgesic Consum. Prediction (%)**	**C4.5 bagging**	**C4.5 AdaBoost**	**C4.5**	ANN^*^	**Random Forest**	**Rotation Forest**	**SVM**^**‡**^	**NB**
Low Consum. Sensitivity	84.3	79.0	76.9	89.2	95.4	84.1	99.9	81.4
Med Consum. Sensitivity	65.8	54.3	51.5	19.1	47.2	60.6	0.0	48.1
High Consum. Sensitivity	47.5	45.1	45.4	8.4	31.8	51.0	0.0	50.8
Low Consum. Precision	81.7	77.4	76.1	62.0	73.2	80.5	52.8	75.4
Med Consum. Precision	60.7	53.6	51.2	20.7	61.4	59.7	0.0	55.6
High Consum. Precision	75.1	52.3	49.3	22.8	85.7	68.2	0.0	51.2
**Overall Accuracy**	**73.1**	**66.1**	**64.1**	**54.7**	**70.6**	**71.7**	**52.7**	**65.4**

^*^ANN consisting of an input layer of 279 input units, one hidden layer of 140 hidden units, and one output layer of 3 output units.

Learning rate= 0.3; momentum rate= 0.2.

^‡^SVM using a radial basis function, exp(−gamma*|u-v|^2^), where gamma=1/(number of attributes)=1/279.

### PCA control readjustment prediction results

As in analgesic consumption prediction, the PCA control readjustment prediction was also based on the first 24 h of PCA usage data, the patient’s physical state, and operation-related attributes. We predicted whether a patient’s PCA control would require readjustment within the following 48 hours. The class ratio of the PCA data was 81% (negative class) to 19% (positive class). The number of patients who needed PCA readjustment was much smaller than the number of patients who did not, creating a class imbalance problem. When classes are imbalanced, conventional learning algorithms often produce classifiers that do little more than predict the most common class. However, the goal of this study is to predict accurately whether any readjustment of PCA settings will be required in later hours. Unlike the evaluation of analgesic consumption prediction, which is based on overall accuracy, the performance of learning strategies with imbalanced data was measured by their true positive rate (i.e., sensitivity), false positive rate, positive predictive value (i.e., precision) and F-score [35]. Overall accuracy is not an appropriate performance measure for prediction in imbalanced data because any predictor that persistently favors the majority class can easily achieve a high overall predictive accuracy. Table 6 lists these performance measures.

**Table 6:** Definitions of performance measures for PCA control readjustment prediction

**Performance Measure**	**Definition**
TPR^a^ (True Positive Rate)	TP/(TP+FN)
FPR (False Positive Rate)	FP/(FP+TN)
Precision^b^	TP/(TP+FP)
F-score	2*TPR*Precision/(TPR+Precision)

^a^True Positive Rate is also known as Sensitivity or Recall.

^b^Precision is also known as Positive Predictive Value.

Because decision tree-based learning performed the best in analgesic consumption prediction, this study only focuses on the analysis of decision tree-based learning in PCA readjustment prediction. We first tested C4.5 with bagging or boosting on the original imbalanced data set, and then applied either under-sampling or over-sampling to balance the classes. We also tested the Random Forest method on the original imbalanced data set, and found similar performance to that of C4.5 with bagging. Both methods had a low true positive rate, false positive rate, and F-score. The distinction between the bagging and Random Forest methods lies in the tree building process. Unlike bagging, Random Forest considers a random subset of attributes during tree construction rather than all the available attributes. The similar performance of these methods corresponds well with the similar characteristics of the bagging [18] and Random Forest methods [33]. To compare the performance of methods combining boosting and synthetic data generation, this study also tests DataBoost-IM [24]. Table 7(a) shows the results. All values were averaged over ten iterations of stratified 10-fold cross validation. Results indicate that class imbalance has a significant effect on bagging and boosting. Without under-sampling or over-sampling, the class imbalance in the PCA data misled both bagging and boosting toward the majority class, as suggested by their low true positive rates. After under-sampling, the bagged C4.5 method achieved the best F-score.

**Table 7:** Results of PCA control adjustment prediction (before and after data cleaning)

**(a) PCA control readjustment prediction (before data cleaning)**										
**PCA Control Prediction (%)**	**C4.5 bagging**	**C4.5 bagging over-sampling**	**C4.5 bagging under-sampling**	**C4.5 AdaBoost**	**C4.5 AdaBoost over-sampling**	**C4.5 AdaBoost under-sampling**	**C4.5**	**Random Forest**	**Rotation Forest**	**DataBoost-IM**
**TPR**	4.3	16.1	41.5	19.6	32.8	47.3	25.5	2.6	12.0	19.7
**FPR**	1.3	9.6	25.6	11.8	23.5	37.1	17.5	0.4	5.0	14.6
**Pos Predict Val**	39.3	28.2	27.5	27.9	24.6	23.1	25.4	38.2	36.3	23.7
**F-score**	**7.5**	**20.2**	**32.9**	**22.5**	**27.9**	**30.9**	**25.2**	**4.7**	**17.6**	**21.4**
**(b) PCA control readjustment prediction (after data cleaning)**										
**TPR**	40.7	51.1	54.4	42.5	55.4	54.0	43.5	31.8	41.8	49.6
**FPR**	22.8	34.0	36.0	30.5	42.1	44.1	33.7	17.3	27.1	36.2
**Pos Predict Val**	29.5	26.1	26.2	24.5	23.6	22.3	23.2	30.2	26.6	24.3
**F-score**	**33.9**	**33.8**	**35.3**	**30.9**	**33.0**	**31.5**	**30.2**	**30.5**	**32.3**	**32.1**

This study also evaluates the proposed nearest neighbor–based data cleaning strategy. After removing “dirty” negative examples from the training data set, we reduced the ratio of negatives to positives from 81:19 to 65:35 on average. Compared with Table 7(a), the results in Table 7(b) demonstrate that this data cleaning strategy improved most of the classifiers significantly (*p*< 0.001) in both true positive rate and F-score. Although the FPR also increased, the F-scores of these learning methods increased significantly (*p*< 0.001), confirming the advantage of this data cleaning strategy.

### Analysis of patient attributes

Because the decision tree algorithm is a divide-and-conquer method, the attributes closer to the root of the decision tree (i.e., at a higher level) are more informative [11]. An analysis of the occurrence frequency of each attribute and its level in the C4.5 bagging trees identified the 10 most informative attributes for the prediction of 72-h total analgesic consumption (i.e., a continuous dose and PCA dose) and the prediction of PCA analgesic consumption only. Most of the informative attributes were related to unit-hour analgesic consumption (e.g., PCA analgesic consumption in the 9^th^ hour (pcadose_9hr)) (Table 8).

**Table 8:** Informative attributes for total analgesic consumption (Continuous + PCA) Prediction

**Attribute**	-log(*p*-val)^*^
contidose_24hr	156.2
contidose_23hr	153.5
contidose_22hr	147.6
pcadose_21hr	9.3
pcadose_19hr	6.3
pcadose_9hr	11.1
pcadose_3hr	15.7
pcadose_2hr	14.4
p_timediff_var_17hr	3.9
pcamode_set_24hr	∞ (*p*-value ≈ 0)

^*^negative logarithm of *p*-value obtained from ANOVA analysis (for pcadose, contidose, and p_timediff_var) or chi-square test (for pcamode_set).

ANOVA analysis (for numeric value attributes) and the chi-square test (for nominal value attributes) were performed to evaluate the correlation between these attributes and symbolic analgesic consumptions (i.e., low, medium, and high). Results show that these attributes were significantly correlated with analgesic consumption. A series of tests was conducted to obtain a baseline *p*-value by randomizing the attributes to further verify the set of informative attributes. Three continuous-dose and five PCA-dose attributes were identified as informative in the 24 unit-hour dose attributes. Thus, we randomly selected three continuous-dose attributes and five PCA-dose attributes in the Monte Carlo tests. The negative logarithms of the average baseline *p*-value were 113.1 (averaged over three random continuous doses) and 10.9 (averaged over five random PCA doses). Compared with the negative logarithms of the average *p*-values of the informative continuous-dose and PCA-dose attributes (i.e., 152.4 and 11.4), these results suggest that these informative attributes were not identified by chance. An examination of the total analgesic consumption of all patients showed that 78.1% of the patients received more volume of analgesia from continuous dose than from PCA dose. This result concurred with the finding that continuous-dose attributes are more significant than PCA-dose attributes (i.e., 152.4 vs. 11.4).

We repeated the same analysis and verification procedures for the prediction results of 72-h PCA analgesic consumption. Table 9 presents a summary of the informative attributes and their ANOVA analysis results. The negative logarithm of the average baseline *p*-value for the PCA dose was 38.4 vs. 44.9, the negative logarithm of the *p*-value averaged over the more important PCA doses (i.e., pcadose_6hr, 9hr, 11hr, 14hr, and 19hr). The negative logarithm of the average baseline *p*-value for the PCA demand time gap means was 10.5 vs. 14.3, the negative logarithm of the average *p*-value of the more informative PCA demand time gap means (i.e., p_timediff_mean_9hr, 14hr, 17hr, and 22hr). These results indicate the significance of the more important and informative attributes identified for the 72-h PCA analgesic consumption prediction.

**Table 9:** Informative attributes for PCA analgesic consumption (PCA only) prediction

**Attribute**	-log(*p*-val)^*^
pcadose_19hr	41.6
pcadose_14hr	47.0
pcadose_11hr	49.0
pcadose_9hr	40.1
pcadose_6hr	46.6
p_timediff_mean_22hr	10.4
p_timediff_mean_17hr	11.3
p_timediff_mean_14hr	16.9
p_timediff_mean_9hr	18.5
p_timediff_var_19hr	5.7

^*^negative logarithm of *p*-value obtained from ANOVA analysis.

Previous research [6,7] has identified a significant correlation between age and the opioid dosage required during the postoperative period. Other studies have reported that gender is an important factor in PCA morphine consumption [8]. To test whether age and gender are important factors for symbolic PCA dose prediction (i.e., “low,” “medium,” and “high,”), this study includes ANOVA analysis and chi-square testing. Results show that age and gender are more significant than other demographic or biomedical attributes, such as ASA class or pulse, in the prediction of PCA analgesic consumption (Table 10). In addition to age and gender, weight was another important attribute. This finding conflicts with previous research showing no correlation between analgesic consumption and patient weight [8,36]. However, epidural-related PCA research has associated body mass index with analgesic requirements [37], suggesting that weight may be a relevant factor.

**Table 10:** Analysis of demographic/biomedical attributes for PCA analgesic consumption prediction

**Attribute**	***p*-value**
age	0.03
gender	0.05
weight	0.0003
sbp	0.37
dbp	0.96
pulse	0.98
ASA CLASS	0.58
OP_CLASS	0.24
op_time	0.10
URGENCY	0.19
ANS_WAY	0.15
DM	0.36
HT	0.45
AMI	0.55

Table 11 shows the top 10 informative attributes for PCA readjustment prediction. Unlike analgesic consumption prediction, this study identifies a wider variety of informative attributes for PCA control adjustment prediction, but ANOVA analysis and the chi-square test showed that some of the informative attributes were not significant (*p*> 0.05). However, the goal of this study is to develop classifiers capable of making accurate and comprehensible predictions rather than simply identifying significant predictive factors, as in most previous research [7-9]. This disagreement in attribute analysis addresses the difference between statistical methods and machine-learning approaches. Systolic blood pressure and pulse were also significant in PCA control readjustment prediction, whereas weight was not. These results conflict with those in PCA analgesic consumption prediction (Table 10), suggesting that these two prediction tasks have different characteristics.

**Table 11:** Informative attributes for PCA control readjustment prediction

**Attribute**	-log(*p*-val)^*^
contidose_24hr	12.0
p_timediff_var_3hr	1.3
p_timediff_var_8hr	0.5
sbp	2.8
pulse	2.1
p_timediff_mean_17hr	0.5
pcamode_set_24hr	3.2
pcamode_set_14hr	2.1
op_time	1.2
weight	1.0

^*^negative logarithm of *p*-value obtained from ANOVA analysis or chi-square test.

PCA is one of the most effective techniques for postoperative analgesia, and is now widely used in hospitals for the management of postoperative pain. To improve patient satisfaction, this study attempts to predict the need for PCA readjustment based on the first few hours of PCA treatment. Based on the PCA patient data provided by CCH, the number of patients that required PCA readjustment was much smaller than those who did not. Learning from imbalanced classes has long been a challenging problem in the machine learning and data mining community. This study of decision tree-based learning evaluates several common approaches to the class-imbalance problem. Under-sampling and over-sampling can both improve the prediction performance of decision tree-based ensemble learning. However, under-sampling outperforms over-sampling in terms of F-score, supporting the hypothesis that data point sparsity blurs the boundary between classes. The PCA data in this study shows that the number of patients that required PCA readjustment was small, and these patients were sparsely distributed in the data space. In this case, over-sampling sparse patients to balance the class size may not sharpen the decision boundary effectively. Over-sampling may also shatter the decision region into many smaller ones (Figure 4(c)), decreasing prediction accuracy owing to overfitting. When the minority class is small and sparse, under-sampling the majority class to balance the classes may be a better approach because it avoids overfitting, even though the decision region is not guaranteed to be of the same class. Like over-sampling, generating artificial data points to balance classes has a similar weakness. Table 7(a) shows that a single C4.5 decision tree outperformed DataBoost-IM with data generation in F-score. This suggests that the data generation process may be misled by the sparse distribution of data points. To mitigate the effects of data sparsity, “dirty” data was removed from the majority class. As expected, the decision regions became more distinct after data cleaning, as demonstrated by the improved F-scores of all the learning methods in this study.

Although PCA can provide medical staff with a convenient way to control pain, it requires constant attention: manually collecting each patient’s PCA data, printing out analgesia usage data, and entering readings into appropriate databases. Based on recent advances in information technology and wireless networking, the objective of information network technology has shifted from increasing hardware performance alone to providing better services and wider applicability. Medical care is one of many potential applications of information network technology. We have combined the Zigbee sensor network and the IEEE 802.11 network to collect and transmit PCA-related data to databases for pain management [38]. Field tests at Changhwa Christian Hospital (CCH) show that the automation of data collection, maintenance, and analysis can significantly reduce the amount of labor work in PCA treatment and increase efficiency.

We are currently developing a 3G-gateway module to further extend the automation of data collection and management. In addition, we plan to connect more medical devices to the sensor network to collect other patient vital signals, such as SpO_2_. Using more patient attributes, the proposed approach should be able to better characterize PCA demand behaviors and make more accurate predictions of PCA analgesic consumption and control adjustment.

## Conclusions

Many factors affect individual variability in postoperative pain. Although several statistical studies have evaluated postoperative pain and analgesic consumption, a systematic review of previous research shows that the coefficient of determination of existing predictive models was small (e.g., *R*^*2*^ = 0.17–0.59 for postoperative pain, and 0.27–0.46 for postoperative analgesic consumption) [39]. These findings indicate that approximately half of the variability is unexplained, and that factors other than demographic or physiological attributes may contribute to the complexity of postoperative outcomes. This study presents the real-world application of data mining to anesthesiology and considers a wider variety of predictive factors, including PCA demands over time. This study analyzes PCA patient data and conducts several experiments to evaluate the potential of applying machine-learning algorithms to assist anesthesiologists in PCA administration. Results confirm the feasibility of the proposed ensemble approach to postoperative pain management.

## Abbreviations

PCA: Patient Controlled Analgesia; ANN: Artificial Neural Network; SVM: Support Vector Machine; NB: Naïve Bayesian; ANOVA: Analysis of Variance; CCH: Changhwa Christial Hospital.

## Competing interests

The authors declare no conflict of interests.

## Authors' contributions

YJH developed the data cleaning method, designed and conducted the experiments, analyzed the results, and drafted the manuscript. THK, RHJ, KW, YCT and SFY collected the data, analyzed the experimental results, and provided feedback on the paper. All authors read and approved the final manuscript.

## Pre-publication history

The pre-publication history for this paper can be accessed here:

http://www.biomedcentral.com/1472-6947/12/131/prepub
